# Quantized Convolutional Neural Networks Robustness under Perturbation

**DOI:** 10.12688/f1000research.163144.1

**Published:** 2025-04-09

**Authors:** Jack Langille, Issam Hammad, Guy Kember

**Affiliations:** 1 Department of Engineering Mathematics and Internetworking, Dalhousie University, Halifax, Nova Scotia, Canada

**Keywords:** Neural network quantization, convolutional neural networks (CNNs), computer vision, model robustness, perturbation modeling, edge AI

## Abstract

Contemporary machine learning models are increasingly becoming restricted by size and subsequent operations per forward pass, demanding increasing compute requirements. Quantization has emerged as a convenient approach to addressing this, in which weights and activations are mapped from their conventionally used floating-point 32-bit numeric representations to lower precision integers. This process introduces significant reductions in inference time and simplifies the hardware requirements. It is a well-studied result that the performance of such reduced precision models is congruent with their floating-point counterparts. However, there is a lack of literature that addresses the performance of quantized models in a perturbed input space, as is common when stress testing regular full-precision models, particularly for real-world deployments. We focus on addressing this gap in the context of 8-bit quantized convolutional neural networks (CNNs). We study three state-of-the-art CNNs: ResNet-18, VGG-16, and SqueezeNet1_1, and subject their floating point and fixed point forms to various noise regimes with varying intensities. We characterize performance in terms of traditional metrics, including top-1 and top-5 accuracy, as well as the F1 score. We also introduce a new metric, the Kullback-Liebler divergence of the two output distributions for a given floating-point/fixed-point model pair, as a means to examine how the model’s output distribution has changed as a result of quantization, which, we contend, can be interpreted as a proxy for model similarity in decision making. We find that across all three models and under each perturbation scheme, the relative error between the quantized and full-precision model was consistently low. We also find that Kullback-Liebler divergence was on the same order of magnitude as the unperturbed tests across all perturbation regimes except Brownian noise, where significant divergences were observed for VGG-16 and SqueezeNet1_1.

## 1. Introduction

Convolutional neural networks (CNNs) have emerged as an effective means of modeling relationships in spatially expressive data, introducing a new domain of computer vision tasks ranging from image classification and segmentation to object detection and video processing. Although impressive in their abilities, contemporary CNNs suffer from increasingly large parameter counts and sophisticated hardware requirements, where parameters are the model’s filter weights and activations, typically stored as 32-bit floating point numbers (FP32). ^4, 12^

In hardware, forward-pass computations are performed by multiply-accumulate (MAC) operations, which incur substantial overhead due to FP32 arithmetic. ^23^ These operations involve manipulating the exponent, mantissa, and special values such as *NaN* (Not a Number) and *infinity*, further increasing memory and computational costs. ^2^ Such limitations are critical in real-time systems like autonomous vehicles, where weight, power consumption, and energy efficiency are tightly constrained. ^9, 3^ Thus, there is motivation to examine methods to reduce the precision of the numeric representation to speed up inference times and reduce hardware and energy requirements.

Quantization has become a popular approach to precision reduction, mapping FP32 weights and activations onto lower-resolution representations such as 8-bit integers (INT8). This technique, commonly used in other domains like digital signal processing for power efficiency and reduced latency, has been successfully adapted for machine learning. ^25^ Prior studies have demonstrated its potential:

1. Vector quantization techniques, such as k-means and product quantization, have achieved up to 24 × compression with minimal accuracy loss. ^13^
2. Compression pipelines combining pruning, quantization, and Huffman coding yielded compression ratios as high as 49 × with significant energy savings. ^16^
3. Optimization methods weighted by parameter importance, such as those based on the Hessian matrix, have further improved quantization efficiency. ^7^

More aggressive strategies, such as binary networks, have also shown promise, with reductions of up to 58 × in computation time and 32 × in memory usage. ^28, 29^ However, these approaches often involve trade-offs in model accuracy and information-storing capacity.

However, given that any precision reduction also decreases a network’s information-storing capacity, it is essential to understand and characterize performance prior to deployment. Specifically, understanding performance in real-world conditions is important to derisk the implementation of quantized networks for practical applications. To this end, this paper experimentally examines the performance of quantized networks under simulated environmental perturbation. We test on four noise regimes, including random additive white Gaussian noise (AWGN), spatially correlated Brownian noise, and vertical and horizontal occlusions. The rationale for these regimes is discussed in later sections.

We also depart from standard benchmark datasets such as MNIST, ^11^ CIFAR, ^20^ and ImageNet ^10^ and study fine-grained visual classification (FGVC). FGVC is relevant to scenarios where it is necessary to predict subclasses within a category, e.g., breeds of dogs or families of aircraft that show high overlap in the representation space. As such, this task generalizes the analysis of datasets such as ImageNet where classes are independent and do not share categorical similarities to such a high degree. Differences between classes in FGVC are comparatively subtle and often are as nuanced as a wing shape or engine mounting, leading to what we contend to be a more substantial assessment of a quantized model’s spatial embedding capacity.

This paper contributes to the quantization literature by addressing the performance of reduced precision networks in FGVC applications through

1. Experimentally evaluating the performance of quantized networks under various forms of perturbation, relative to their full-precision counterparts,
2. Experimentally evaluating quantized networks ability to perform fine-grained visual classification,
3. Employing Kullback-Leibler (KL) divergence as a metric to quantify the similarity in decision making between quantized and full-precision models.

These results serve as a transferable contribution to understand robustness of quantized networks to perturbations in the analysis of subclass datasets (e.g. FGVC) where the representation shows high overlap.

## 2. Methods

### 2.1 Quantization

In this work we adhere to common quantization schemes with neural networks and map the higher-resolution set of possible FP32 numbers to a lower-resolution subset of INT8 numbers. ^23, 35^ In accordance with typical approaches to network quantization, we are quantizing the weights and outputs (activations) through the network ^23, 35, 37^ We also consider strictly post-training quantization (PTQ) rather than quantization-aware training (QAT), as it does not require a complete re-training step but a simple calibration step on unlabeled data. While generally QAT yields higher compression rates and lower error rates, ^23^ as this paper will show, PTQ still performs well and comes with the benefit of faster and less resource-intensive implementation.

PTQ necessitates first defining several key parameters: the bit-width b , the step-size or scale factor, s , and the zero-point z _._^23^ The bit-width defines the number of possible levels in the quantization grid. The scale factor sets the step-size or difference between each level. The zero-point is an integer chosen such that the actual zero is quantized without error and is important to ensure activation functions like ReLU do not introduce additional quantization errors. ^23^

Using symmetric or affine quantization involves mapping parameters to the quantization grid depending on the symmetry of the scheme. For the unsymmetric (affine) case, we have

$$x_{\mathrm{INT}8}=\text{clamp}\left(\lfloor \frac{x_{\mathrm{FP}32}}{s}\rceil +z{,0,2}^{b}-1\right).$$ (1)

For the symmetric about z case

$$x_{\mathrm{INT}8}=\text{clamp}\left(\lfloor \frac{x_{\mathrm{FP}32}}{s}\rceil +z,-2^{b-1},2^{b-1}-1\right).$$ (2)

The notation ⌊⋅⌉ is the round-to-nearest integer operator while the clamping function is defined as

$$\text{clamp}\left(x;a,c\right)=\left\{ \begin{array}{ll} a & \text{if}\;x<a,\\ x & \text{if}\;a\leq x\leq c,\\ c & \text{if}\;x>c, \end{array}\right.$$ (3)

and the parameters a and c denote the bounds of the integer grid. The quantization range is bounded by $q_{\min}$ and $q_{\max}$ , and these are defined in terms of quantization symmetry. For affine quantization

$$q_{\min}=-\mathrm{sz}$$ (4)

$$q_{\max}=s\left(2^{b}-1-z\right).$$ (5)

For the symmetric case, z is constrained to 0, and the range is bounded by

$$q_{\min}=-s\left(2^{b-1}\right),$$ (6)

$$q_{\max}=s\left(2^{b-1}-1\right).$$ (7)

As mentioned, we select a bit width of 8-bits, the most commonly used scheme to balance compression and error rates. ^35^ Scale and zero point are determined using optimization algorithms packaged with PyTorch’s ^26^ quantization library. We quantize weights symmetrically and the activations in an affine fashion. These choices reflect that weights are approximately symmetrically distributed around 0, whereas activations are positively skewed owing to their activation function, e.g., ReLU.

### 2.2 Dataset

**Figure 1.**
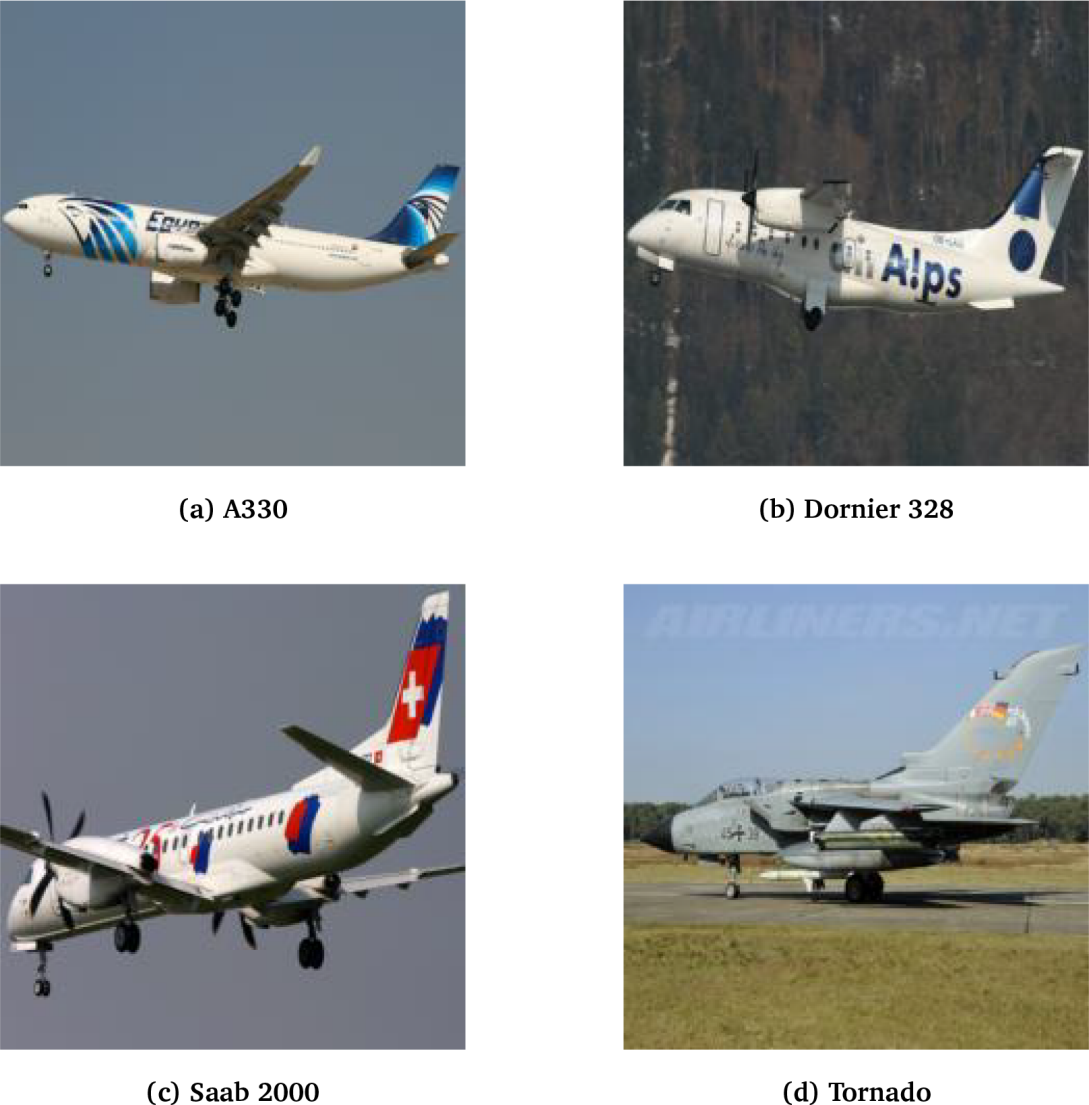
Sample imagery from the FGVC-A dataset.

To study FGVC, we use the fine-grained visual classification aircraft (FGVCA) dataset. ^22^ FGVCA was first introduced as part of the 2013 International Conference on Computer Vision (ICCV), and since then has seen frequent use in the literature. ^34, 5^ FGVCA is comprised of 10000 1-2 Mpixel images and provides several classification tasks and labels, including aircraft model (most specific), variant, family, and manufacturer (least specific). ^22^ The family classification task is considered here and comprises 70 family labels. Examples of families include Boeing-737, which includes variants like 737-200 or 737-300. ^22^ Figure 1 illustrates several dataset samples. Dataset images have varying resolutions and aspect ratios and are altered to a consistent input size as part of the pre-processing transformation.

Other FGVC datasets are available, including natural species, ^8^ birds, ^18^ or flowers. ^24^ FGVCA has important subclass properties where aircraft features are strongly dependent on size (hobbyist aircraft to large transport aircraft), purpose (commercial, pleasure, or military), and technology (turbine propulsion, propeller, glider, etc.). Each of these designs have unique structural features such as the wing shape and size, fuselage style, landing gear/wheels, and engine mounting. Another interesting feature of FGVCA is that different organizations such as airlines and military often have slight modifications such as branding and camouflage, meaning classes can have the same rigid structure, but distinctly different "looks". Cumulatively, these features result in rigid variability across classes compared to tasks such as birds or flowers, where non-trivial variations can exist within the same class due to mutations, climate, etc. This rigidity is important for our study as it ensures that errors owing to ambiguity in classes are minimized, allowing us to focus exclusively on errors owing to each model’s capacity for spatial embedding.

### 2.3 Perturbations

To model real-world phenomena, we selected additive white Gaussian Noise (AWGN), spatially correlated Brownian noise, and vertical and horizontal occlusions. AWGN is commonly used as a model for thermal noise experienced in circuits and random photo-sensor noise. ^14, 6^ Additionally, AWGN is commonly used in adversarial attacks against deep learning-based systems. ^30, 38^ Brownian noise approximates correlated noise like smoke, fog, clouds, and underwater distortions. Vertical and horizontal occlusions are a model of structured image corruption owing to losses from sensor malfunctions, data corruption during transmission, or solar radiation resulting in rows or columns of dead pixels.

Given an image, **x**:

$$x\in {\mathbb{R}}^{H\times W\times C}$$ (8)

with height H , with W , and channels C , we our perturbations are defined as follows. In AWGN, each pixel’s noise term is independently and identically sampled from a single Gaussian distribution. The lack of correlation between noise samples yields a grainy image. Given the univariate Gaussian density function with mean μ=0 and variance $\sigma^{2}$ :

$$N\left(0,\sigma^{2}\right)=\frac{1}{\sqrt{2{\pi \sigma }^{2}}}e^{-\frac{{\left(x\right)}^{2}}{2\sigma^{2}}}.$$ (9)

Pixels are indexed by i,j, and k respectively denoting row, column and channel yielding our C channel additive noise mask $n_{g}\left(i,j,k\right)$ :

$$n_{g}\left(i,j,k\right)∼N\left(0,\sigma^{2}\right).$$ (10)

The perturbed input follows from a simple addition of the noise mask to the original input:

$$X^{\prime }\left(i,j,k\right)=X\left(i,j,k\right)+n_{g}\left(i,j,k\right).$$ (11)

**Figure 2.**
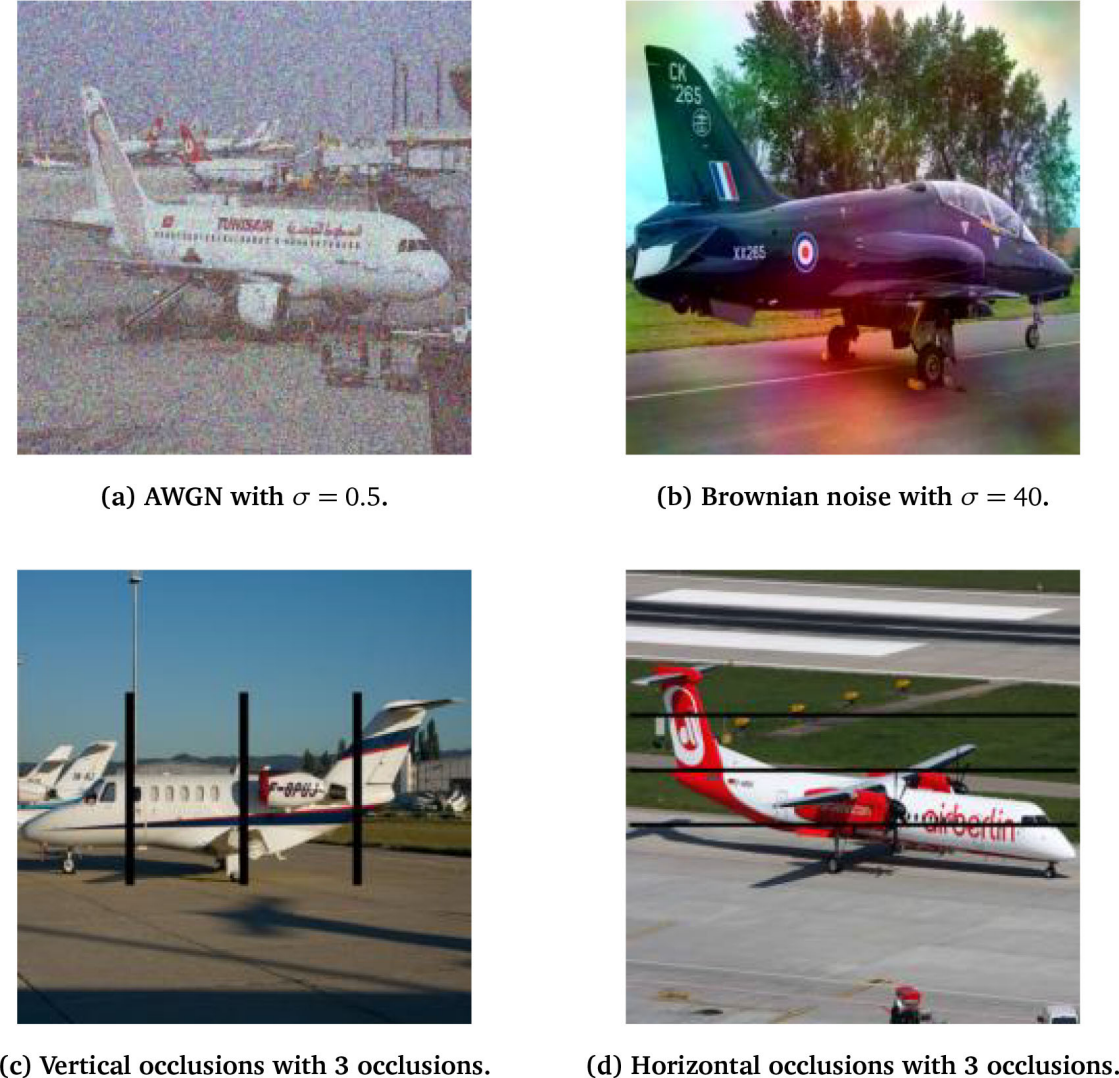
Sample imagery from the FGVC-A dataset under various perturbations.

Brownian noise differs from AWGN in that it is *not* independent and identically distributed, but rather spatially correlated. Moreover, unlike the flat power spectral density of AWGN, Brownian noise has a power spectral density proportional to the inverse square of the frequency, $\frac{1}{f^{2}}$ , and lower frequencies dominate the noise spectrum. Thus, lower frequencies are amplified while higher frequencies are attenuated, giving long-range, smoothly-varying spatial correlations. Brownian noise adds blotches and blur-like artifacts to an image as illustrated in Figure 2.

To generate Brownian noise, we start with white noise, $n_{g}\left(i,j,k\right)$ , sampled from (9). These noises are fast Fourier transformed (FFT) for each of the three channels and yield the frequency components u,v,z , or W(u,v,z) :

$$W\left(u,v,z\right)=\mathrm{FFT}\left[n_{g}\left(i,j,k\right)\right].$$ (12)

We then scale each frequency component by $\frac{1}{f^{2}}$ , where f is the frequency magnitude, $\sqrt{u^{2}+v^{2}+z^{2}}$ :

$$W^{\prime }\left(u,v,z\right)=\frac{W\left(u,v,z\right)}{u^{2}+v^{2}+z^{2}}.$$ (13)

Finally, we apply the inverse FFT (IFFT) to convert to the spatial domain and add the resulting noise to the image:

$$n_{b}\left(i,j,k\right)=\text{IFFT}\left[W^{\prime }\left(u,v,z\right)\right],$$ (14)

$$X^{\prime }\left(i,j,k\right)=X\left(i,j,k\right)+n_{b}\left(i,j,k\right).$$ (15)

Lastly, we examine highly structured noise in vertical and horizontal black-out occlusions, or streaks, applied within a pre-defined bounding box of the classification target within each image, as shown in Figure 2. We generate these as having constant width such that increasing the number of streaks increases the image coverage. We control the intensity or degree of perturbation induced by such occlusions by adjusting the number of streaks present in the class’ bounding box region. Edges introduced by occlusions are expected to significantly degrade performance since CNNs extract features such as edges from images, and the occlusions may cause irrelevant feature activation and false edge identification. Streaks may also cover important or distinguishing features of a target class, such as its engine or wing structures, and reduce network performance.

### 2.4 Kullback-Liebler divergence

To extend our analysis of the performance of quantized networks to the softmax probability distribution, we study the KL divergence between the output probabilities of each FP32 and INT8 model. KL divergence, introduced in, ^1^ quantifies the difference between two distributions P (true) and Q (baseline). ^32^

$$D_{\mathrm{KL}}\left(P∥Q\right)=\sum_{i}P\left(i\right)\log\frac{P\left(i\right)}{Q\left(i\right)},$$ (16)

and i is the number of possible states. In our context, we wish to compare the divergence of an INT8 quantized model’s output class probabilities to its full-precision counterpart, given K output classes. It is asymmetric, i.e., $\left(D_{\mathrm{KL}}\left(P∥Q\right)\neq D_{\mathrm{KL}}\left(Q∥P\right)\right)$ , and always non-negative, reaching zero if and only if P and Q are identical. We denote the INT8 and FP32 models’ class probability distributions as $P_{\mathrm{INT}8}$ and $P_{\mathrm{FP}32}$ and then

$$D_{\mathrm{KL}}\left(P_{\mathrm{FP}32}∥P_{\mathrm{INT}8}\right)=\sum_{k=1}^{K}P_{\mathrm{FP}32}\left(k\right)\log\frac{P_{\mathrm{FP}32}\left(k\right)}{P_{\mathrm{INT}8}\left(k\right)}.$$ (17)

If we observe similar accuracy with high KL divergence, the quantized model maintains accuracy at the cost of what is termed here *confidence.* For example, consider an FP32 model that yields the following output probabilities: class 1: 0.7, class 2: 0.2, class 3: 0.1, and an INT8 model that yields class 1: 0.5, class 2: 0.4, class 3: 0.1, where the actual label is class 1. Both models correctly classified class 1 as the actual label. However, they have markedly different degrees of confidence. Of course, KL divergence alone does not grant insight into class-by-class probabilities, but it does quantify a macro-level model similarity, which may serve as a precursor to specific class probability studies. However, it is important to recognize that this metric assumes the FP32 model is the *true* baseline distribution. We contend that this is a fair characterization as, in reality, it is the theoretical *best* we can do regarding capacity for information embedding per parameter. In this case, the baseline is indeed dynamic in the sense that the distribution of the FP32 model will change under a perturbation. Given that both the FP32 and INT8 models are tested on identical inputs with identical perturbation, we are then answering the question *under perturbation level X, how does our quantized model deviate in its probabilities from what would otherwise be outputted given no quantization?*

## 3 Experimental procedure

### 3.1 Models and training

We examine three state of the art models including VGG-16, ^33^ ResNet-18, ^17^ and SqueezeNet1_1. ^19^ These models were selected on the basis of their varying size and ubiquity in the current body of computer vision research. We compare the size of each network in terms of parameter count and floating point operations (FLOPs) in Table 1. Models were downloaded from PyTorch’s model zoo, ^26^ with pre-trained weights for ImageNet. ^10^ Each model was adjusted to have 70 output neurons, in accordance with the FGVCA families classification task. Models were trained for 250 epochs on a training set of shuffled 3333 images, using the cross-entropy loss function to measure the prediction error. The stochastic gradient descent optimizer was employed with a learning rate of 0.001 and a momentum of 0.9 to update the model parameters during training. Additionally, a learning rate scheduler was applied to decrease the learning rate by a factor of 0.1 every 50 epochs.

**Table 1.** Details of studied models. All data retrieved from PyTorch documentation. ^26^

Model	Parameter (M)	Size (MB)	GFLOPs
VGG-16	138.4	527.8	15.47
ResNet-18	11.7	44.7	1.81
SqueezeNet1_1	1.2	4.7	0.35

### 3.2 Perturbation intensities

We examine the performance of each model under each of the perturbations discussed above. As noted, each of these perturbations has its own respective intensity parameter. For AWGN and Brownian noise it is standard deviation, and the number of occlusions for vertical and horizontal occlusions. In each case, the intensity range was experimentally selected to best capture the full spectrum of degradation, as is shown in the accuracy/F1 plots in Section 3. For AWGN we study standard deviations ranging from 0 to 1 with a step size of 0.1 from 0.1 to 0.6, and then 0.2 from 0.6 to 1.0 as beyond 0.6 model performance plateaus. For Brownian noise we study standard deviations ranging from 10 to 80 with a step size of 10. For vertical and horizontal occlusions we select occlusions ranging from 1 to 6 with step sizes of 1. Note that the inclusion of even 1 streak yielded significant degradation across all metrics, and as more occlusions were added, performance quickly degraded to near 0 across all metrics. Also, as discussed in Section 1.3 the Brownian perturbation regime required comparatively higher standard deviations than AWGN to yield any noticeable degradation. This is expected, as Brownian noise is smoother and more structured than AWGN, owing to its $1/f^{2}$ spectrum, making it less disruptive to the image’s overall structure and features when compared to AWGN’s direct pixel-by-pixel variations. For each perturbation scheme each model’s FP32/INT8 pair were tested on the full test set of 3333 images.

### 3.3 Metrics computation

We are interested in computing 4 primary metrics, top-1 accuracy, top-5 accuracy, F1-score, and KL divergence. Top 1 accuracy, top 5 accuracy, and F1 score are computed using Scikit-learn. ^27^ To compute KL divergence we store the 70 class output probabilities for each model pair and compute the KL divergence between them, on a per image basis, for each perturbation level. We then compute the average KL divergence across the entire test set of 3333 images, for each perturbation level, resulting a in a single scalar averaged measure for each model pair.

## 4. Results and Discussion

### 4.1 Unperturbed results

We begin by examining the unperturbed results to establish a baseline. Table 2 presents F1, top-1 and top-5 accuracies for each model pair with VGG-16 showing the best performance across categories and SqueezeNet1_1 the worst. This result is expected result and matches the general trend presented in Table 1 which supports the notion that increased parameter count driven by layer depth yields higher accuracy. As expected, we can see that top-1 accuracy is comparatively worse than top-5 accuracy across all models - SqueezeNet1_1 exhibits a 14% top-1 accuracy drop compared to VGG-16 but only a 6% drop in top-5 accuracy. This is indicative of the nature of FGVC where it is likely that smaller models like SqueezeNet1_1 lack the expressive power to capture the subtleties between similar classes but can make a *near* correct prediction. We also see that F1 - the average of a model’s ability to avoid false positives (precision) and false negatives (recall) - follows the same trend as top-1 accuracy. Most notable, however, is that we observe very little degeneration in model performance across all three metrics post-quantization. This result is congruent with ^23, 35, 37^ and confirms that our quantization scheme is implemented and performing as expected. Table 3 presents KL divergences for each model pair. Interestingly, ResNet-18 exhibits the *lowest* KL divergence by an order of magnitude, and VGG-16 the highest. While all relatively low, these results may reflect each model’s architecture. For instance, ResNet-18’s skip connections, may aid in mitigating quantization error propagation where VGG-16’s depth without such connections may exacerbate quantization error propagation.

**Table 2.** Baseline unperturbed top-1 and top-5 accuracies.

	SqueezeNet1_1		ResNet-18		VGG-16	
Metric	FP32	INT8	FP32	INT8	FP32	INT8
Top-1 Acc	0.6268	0.6250	0.7204	0.7192	0.7843	0.7828
Top-5 Acc	0.8866	0.8866	0.9241	0.9256	0.9487	0.9475
F1 Score	0.6287	0.6268	0.7153	0.7145	0.7835	0.7828

**Table 3.** Baseline unperturbed KL divergences.

Model Pair	KL Divergence
SqueezeNet1_1	0.0138
ResNet-18	0.0082
VGG-16	0.0182

### 4.2 Perturbed results

**Figure 3.**
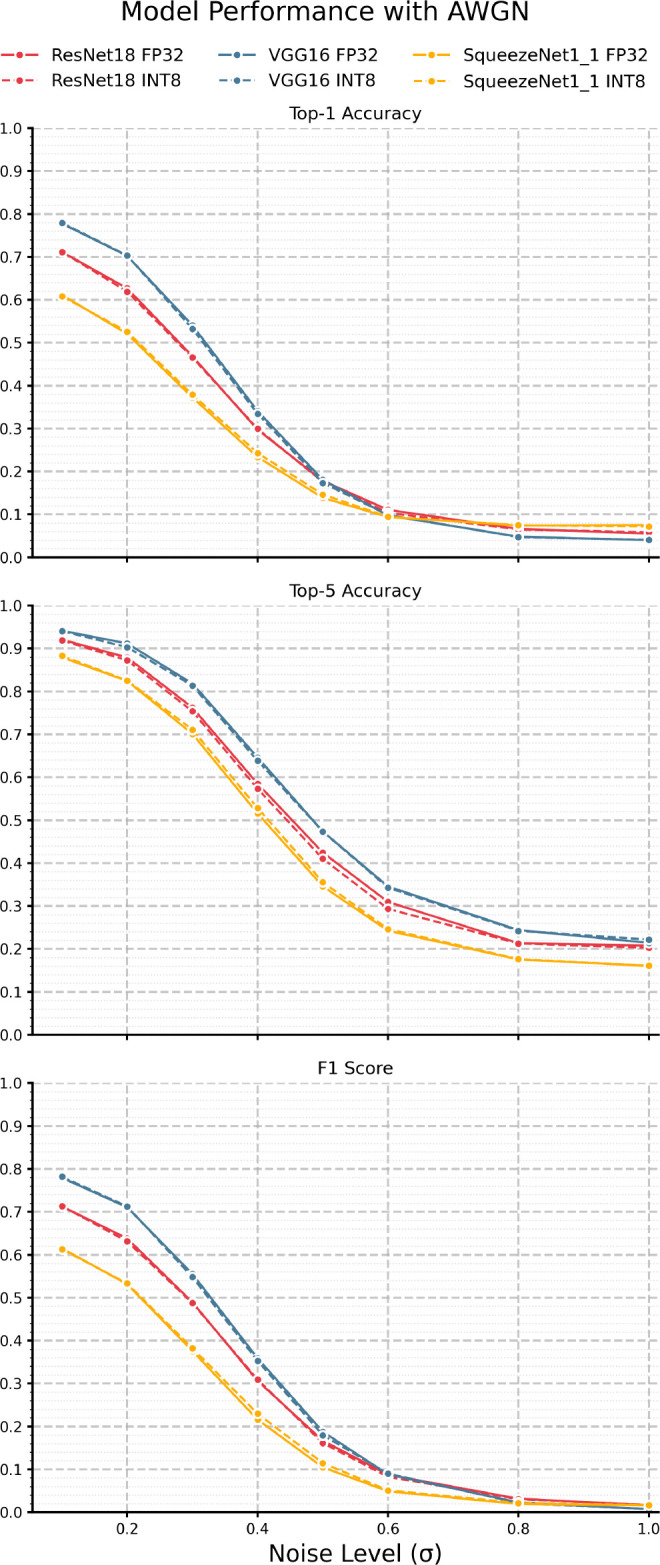
Performance of each model pair under varying levels of AWGN.

We begin by looking at the impacts of AWGN, presented in Figure 3, and Table 4. Across all models and metrics, we see steep degeneration beginning at σ = 0.2 and plateauing at σ = 0.8, indicating a point of potential ill-conditioning in this particular noise regime. As expected, top-5 accuracy is consistently higher than top-1 by a 20% - 30% margin, with the gap between them widening as noise levels increase. This result suggests that while the exact classification becomes more challenging, the correct class will likely remain among the top 5 predictions. Moreover, in line with our baseline observations, VGG-16 generally outperforms ResNet-18 and SqueezeNet1_1, and ResNet-18 typically outperforms SqueezeNet1_1, with the gap narrowing at higher noise levels. F1 scores follow similar trends to accuracy metrics but show a steeper decline with respect to noise, suggesting that both precision and recall are possibly more severely impacted by noise than accuracy metrics. Most important to this study, however, is that in all cases, we see no significant divergence in performance curves between each FP32/INT8 model pair, directly supporting the notion that quantized models, under AWGN, are *just as* performant, according to accuracy and F1 metrics, irrespective of *overall* losses. Figure 5 and Table 6 present the KL divergences under AWGN. Here, we witness interesting non-linear behavior - under AWGN, each model’s INT8 representation exhibits peak divergence in its class output probabilities in the σ = 0.4-0.6 range. Also, in line with the trend in baseline KL divergences, VGG-16 demonstrates the highest divergence despite being less sensitive in its accuracy and F1 response curves. This suggests that while VGG-16 in its INT8 form is demonstrably robust according to accuracy and F1, its output distribution shifts from its FP32 form, potentially indicating decreased *overall* confidence or *misplaced* confidence. This result is particularly relevant in threshold-based systems where decisions are made based on some output probability threshold. The drop in KL divergence after σ = 0.6 is most likely due to the model pairs reaching a saturation point in error, where, in effect, each model pair is equally weak and unable to make any meaningful predictions, which is evidenced in the accuracy plots where we can see sub-20% top-1 accuracy and sub-40% top-5 accuracy for each quantized and full-precision model.

**Table 4.** AWGN performance results.

	Top-1 Accuracy						Top-5 Accuracy						F1 Score					
σ	SqueezeNet1_1		ResNet18		VGG16		SqueezeNet1_1		ResNet18		VGG16		SqueezeNet1_1		ResNet18		VGG16	
	FP32	INT8	FP32	INT8	FP32	INT8	FP32	INT8	FP32	INT8	FP32	INT8	FP32	INT8	FP32	INT8	FP32	INT8
0.1	0.611	0.608	0.712	0.712	0.778	0.779	0.879	0.883	0.921	0.919	0.941	0.941	0.615	0.613	0.713	0.713	0.779	0.782
0.2	0.521	0.526	0.627	0.619	0.702	0.703	0.824	0.825	0.879	0.872	0.912	0.903	0.531	0.533	0.638	0.631	0.711	0.713
0.3	0.372	0.379	0.469	0.466	0.541	0.532	0.701	0.711	0.762	0.754	0.818	0.814	0.376	0.382	0.489	0.487	0.555	0.548
0.4	0.234	0.243	0.297	0.300	0.341	0.335	0.516	0.529	0.585	0.573	0.646	0.638	0.216	0.230	0.306	0.309	0.359	0.353
0.5	0.139	0.146	0.178	0.176	0.181	0.173	0.346	0.356	0.425	0.410	0.474	0.473	0.105	0.115	0.167	0.161	0.188	0.180
0.6	0.094	0.095	0.111	0.104	0.098	0.099	0.242	0.246	0.310	0.293	0.346	0.343	0.049	0.051	0.087	0.083	0.088	0.090
0.8	0.074	0.075	0.066	0.065	0.048	0.047	0.176	0.176	0.214	0.213	0.244	0.242	0.019	0.022	0.032	0.030	0.023	0.021
1.0	0.075	0.072	0.055	0.059	0.040	0.041	0.161	0.161	0.208	0.203	0.214	0.222	0.016	0.017	0.016	0.017	0.007	0.007

**Figure 4.**
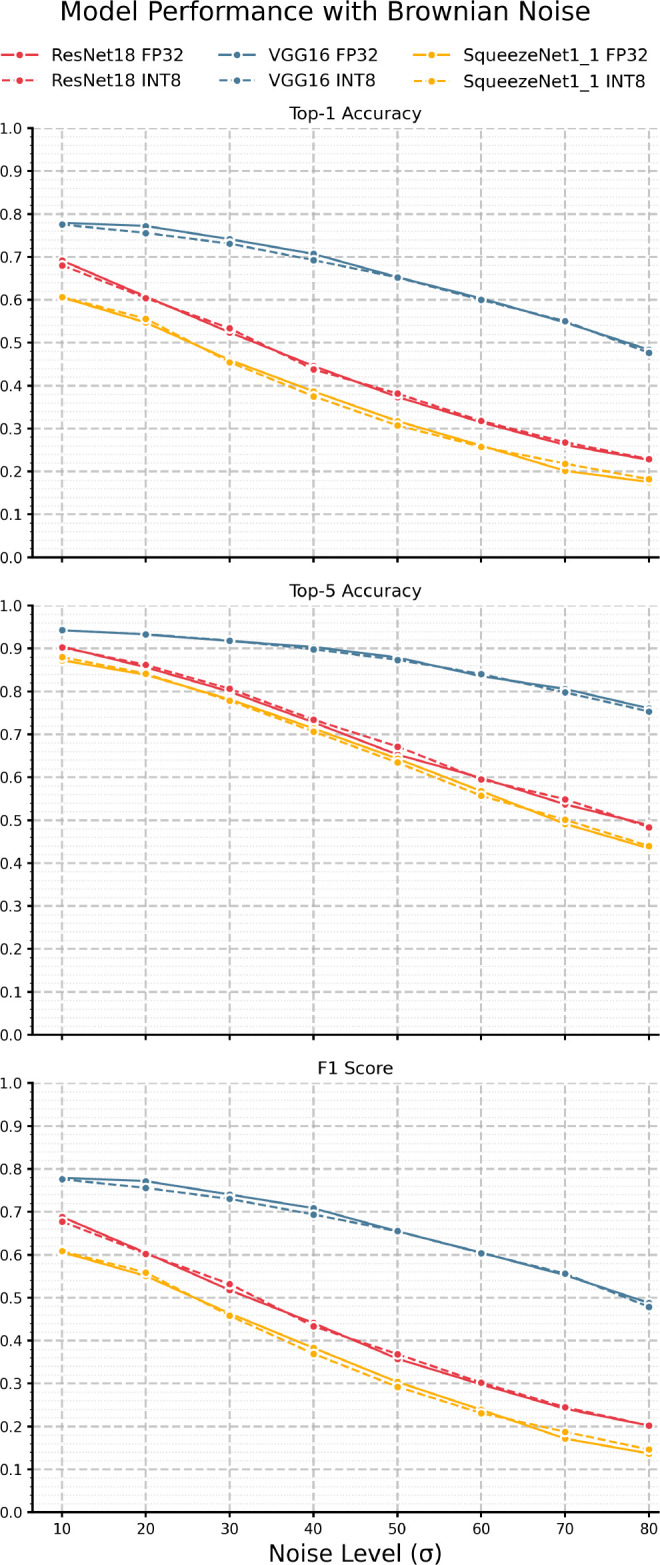
Performance of each model pair under varying levels of Brownian noise.

**Figure 5.**
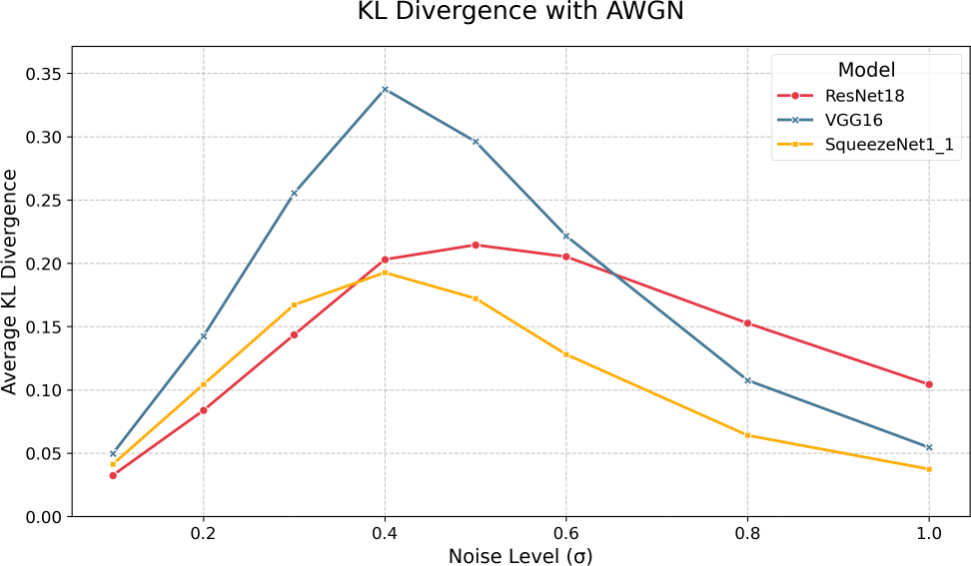
KL divergences for each model pair under various levels of AWGN.

**Table 5.** Brownian Noise Performance Results.

	Top-1 Accuracy						Top-5 Accuracy						F1 Score					
σ	SqueezeNet1_1		ResNet18		VGG16		SqueezeNet1_1		ResNet18		VGG16		SqueezeNet1_1		ResNet18		VGG16	
	FP32	INT8	FP32	INT8	FP32	INT8	FP32	INT8	FP32	INT8	FP32	INT8	FP32	INT8	FP32	INT8	FP32	INT8
10	0.606	0.607	0.692	0.680	0.779	0.776	0.873	0.880	0.905	0.902	0.943	0.943	0.606	0.608	0.688	0.677	0.779	0.776
20	0.547	0.556	0.608	0.604	0.772	0.756	0.839	0.842	0.856	0.862	0.933	0.933	0.550	0.559	0.604	0.602	0.772	0.756
30	0.460	0.455	0.524	0.533	0.741	0.731	0.782	0.778	0.800	0.806	0.917	0.919	0.463	0.458	0.518	0.532	0.740	0.730
40	0.387	0.375	0.446	0.438	0.707	0.692	0.714	0.707	0.728	0.734	0.904	0.898	0.383	0.369	0.441	0.433	0.708	0.694
50	0.317	0.307	0.374	0.382	0.653	0.652	0.643	0.634	0.653	0.672	0.879	0.874	0.304	0.292	0.358	0.369	0.655	0.655
60	0.260	0.258	0.315	0.318	0.603	0.600	0.568	0.557	0.599	0.595	0.836	0.841	0.239	0.231	0.298	0.302	0.605	0.604
70	0.202	0.218	0.262	0.268	0.547	0.550	0.491	0.501	0.537	0.549	0.806	0.798	0.172	0.188	0.241	0.245	0.552	0.556
80	0.176	0.182	0.227	0.229	0.484	0.476	0.434	0.440	0.488	0.484	0.761	0.753	0.137	0.146	0.202	0.202	0.488	0.479

**Table 6.** AWGN KL Divergences.

σ	SqueezeNet1_1	ResNet18	VGG16
0.1	0.041	0.032	0.050
0.2	0.104	0.084	0.142
0.3	0.167	0.144	0.255
0.4	0.193	0.203	0.338
0.5	0.172	0.214	0.296
0.6	0.128	0.205	0.222
0.8	0.064	0.153	0.108
1.0	0.037	0.104	0.055

**Figure 6.**
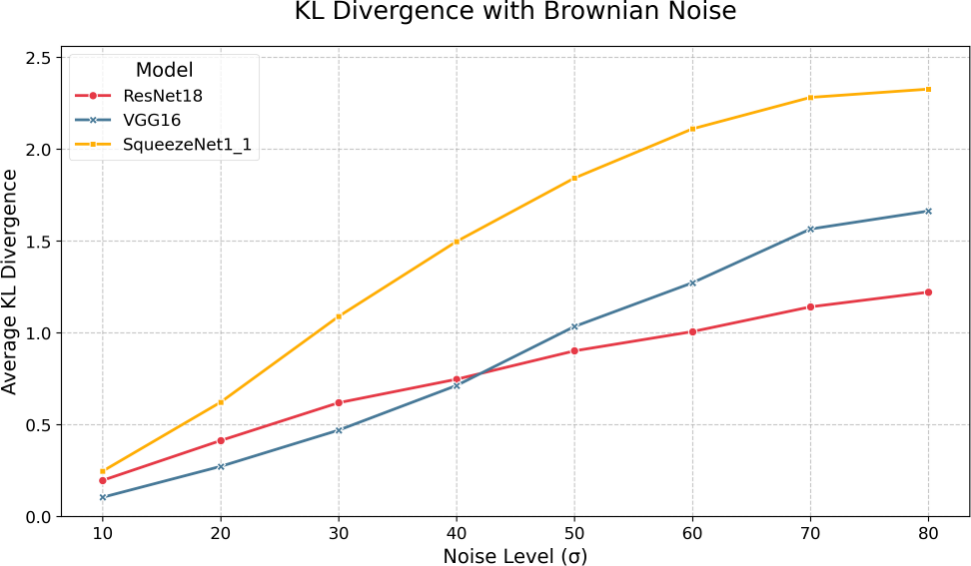
KL divergences for each model pair under various levels of Brownian noise.

Next, we examine the impacts of varying levels of Brownian noise. Figure 4 and Table 5 present the F1 scores, top-1 and top-5 accuracies where we can see very different response curves compared to the AWGN results. It is clear that, as expected, the effects of Brownian noise are much more gradual compared to AWGN, as the spatial correlation of Brownian noise yields a much smoother noise pattern. Like AWGN, we see VGG-16 outperform all models as it maintains top-1 accuracy above 50% and top-5 accuracy above 70% until σ = 50. Interestingly, we can see that Brownian noise seems to induce a relatively large error in the quantized model from the FP32 model compared to AWGN, especially in VGG-16 in the σ = 10-50 range. In terms of F1, we also observe more significant discrepancies between the quantized and full-precision models; for instance, VGG-16 exhibits roughly 2% error in F1 at σ = 20. Also, as expected, the top-5 accuracy is consistently higher than the top-1 accuracy in all three models. KL divergence, as shown in Figure 6 and Table 7, is much higher than AWGN and does not have the same peaking behavior shown in Figure 5 - instead we see a steady increase with noise across all three models. Like AWGN, VGG-16 exhibits the highest divergence, again suggesting a comparatively higher discrepancy in the model’s confidence among output classes when making predictions. ResNet-18, in contrast, yields the lowest divergence. Overall, we can see that the impacts of Brownian are much more gradual; however, they induce significantly higher KL divergences when compared to AWGN.

**Table 7.** Brownian noise KL divergences.

σ	SqueezeNet1_1	ResNet18	VGG16
10	0.247	0.197	0.105
20	0.622	0.413	0.273
30	1.089	0.619	0.470
40	1.498	0.747	0.713
50	1.843	0.902	1.035
60	2.111	1.007	1.273
70	2.282	1.142	1.565
80	2.327	1.222	1.663

**Figure 7.**
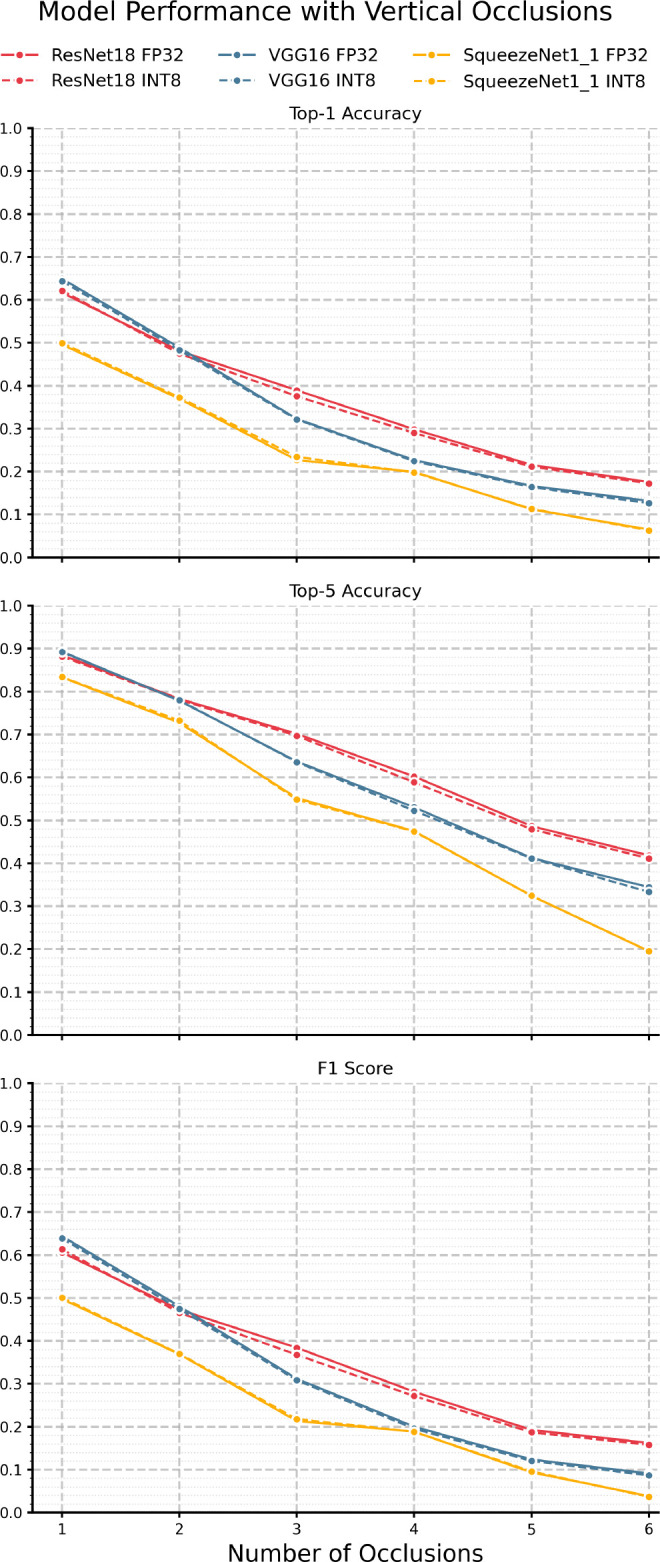
Performance of each model pair under various amounts of vertical occlusion.

We now look at the results under vertical occlusions, given in Figure 7 and Table 8. Here, we see performance degradation in accuracy and F1 gradually dropping off, similar to Brownian noise, which is an expected result as occlusions are another form of highly correlated noise. However, while the decrease is gradual, the immediate drop off at just 1 occlusion is significant. For example, the inclusion of 2 vertical occlusions yielded sub-50% top-1 accuracy across all models. With respect to quantization, we see the same general trend as other perturbation regimes, in that there is minimal divergence between each FP32/INT8 model pair, again suggesting that performance degradation is a *macroscopic* phenomenon - a function of model architecture and overall size, not parameter-wise information capacity. Indeed, each model exhibits relatively low KL divergence, with the max being 0.0636 for VGG-16 at two vertical occlusions, as shown in Figure 9 and Table 10. Moreover, the lack of divergence is relatively constant, with the most significant delta being just 0.038 for VGG-16 between 1 and 3 occlusions. However, it is notable that ResNet-18 exhibits the lowest KL divergence, which, paired with its performance in top-1, top-5, and F1, indicates it is comparatively robust against highly structured perturbation.

**Table 8.** Vertical occlusions performance results.

	Top-1 Accuracy						Top-5 Accuracy						F1 Score					
Num.	SqueezeNet1_1		ResNet18		VGG16		SqueezeNet1_1		ResNet18		VGG16		SqueezeNet1_1		ResNet18		VGG16	
	FP32	INT8	FP32	INT8	FP32	INT8	FP32	INT8	FP32	INT8	FP32	INT8	FP32	INT8	FP32	INT8	FP32	INT8
1	0.496	0.500	0.617	0.622	0.649	0.644	0.833	0.834	0.885	0.882	0.893	0.892	0.498	0.501	0.606	0.613	0.644	0.639
2	0.370	0.373	0.481	0.475	0.489	0.483	0.727	0.732	0.783	0.781	0.779	0.780	0.369	0.370	0.473	0.466	0.481	0.474
3	0.227	0.234	0.389	0.376	0.323	0.321	0.552	0.548	0.702	0.697	0.637	0.636	0.213	0.218	0.384	0.368	0.312	0.308
4	0.199	0.198	0.299	0.290	0.227	0.224	0.475	0.474	0.602	0.589	0.530	0.523	0.189	0.188	0.281	0.271	0.200	0.195
5	0.112	0.113	0.216	0.212	0.167	0.164	0.324	0.325	0.487	0.479	0.413	0.411	0.094	0.096	0.192	0.187	0.124	0.120
6	0.065	0.063	0.176	0.173	0.132	0.126	0.195	0.195	0.418	0.411	0.344	0.333	0.038	0.037	0.162	0.158	0.091	0.087

**Figure 8.**
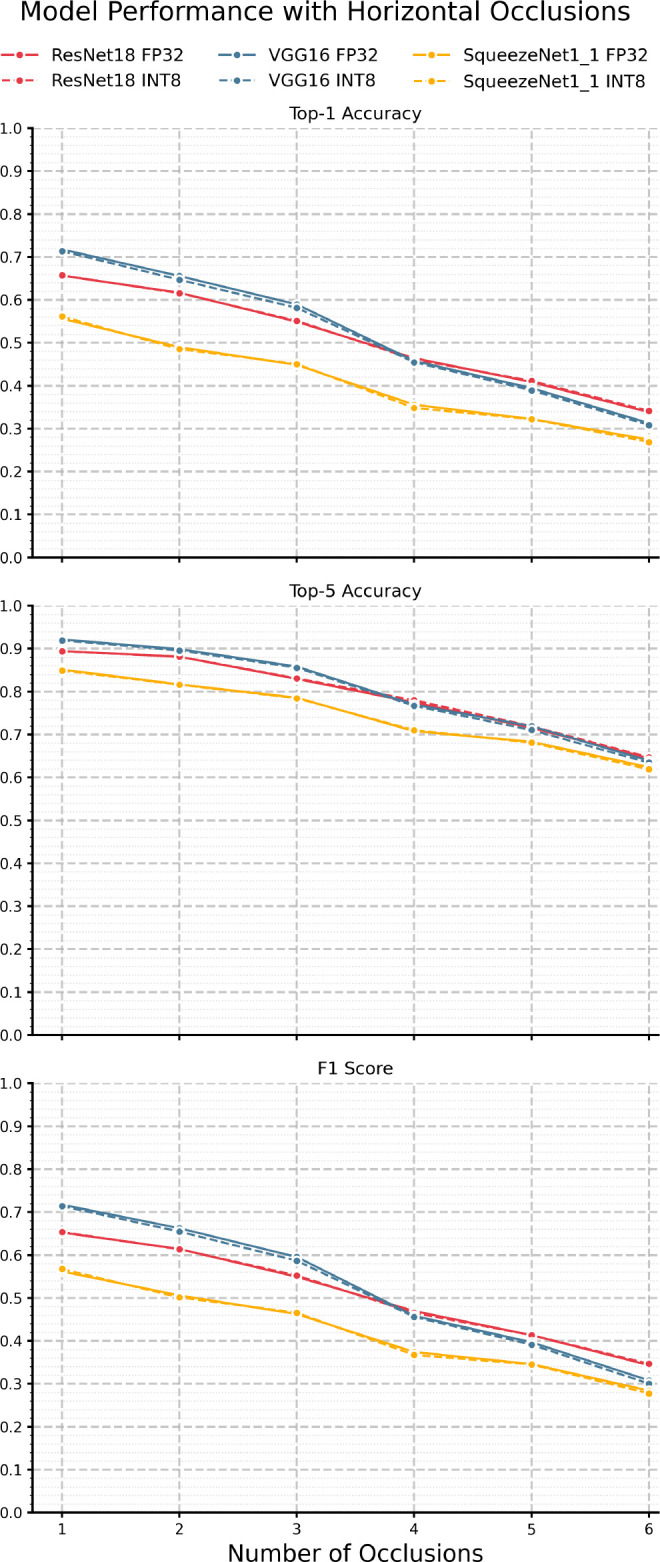
Performance of each model pair under various amounts of horizontal occlusion.

**Figure 9.**
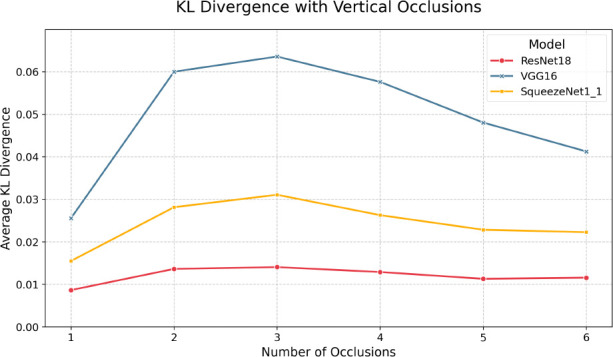
KL divergences for each model pair under various amounts of vertical occlusion.

**Table 9.** Horizontal occlusions performance results.

	Top-1 Accuracy						Top-5 Accuracy						F1 Score					
Num.	SqueezeNet1_1		ResNet18		VGG16		SqueezeNet1_1		ResNet18		VGG16		SqueezeNet1_1		ResNet18		VGG16	
	FP32	INT8	FP32	INT8	FP32	INT8	FP32	INT8	FP32	INT8	FP32	INT8	FP32	INT8	FP32	INT8	FP32	INT8
1	0.556	0.561	0.656	0.657	0.718	0.713	0.851	0.849	0.894	0.894	0.921	0.920	0.562	0.567	0.652	0.653	0.717	0.714
2	0.490	0.485	0.617	0.616	0.656	0.647	0.817	0.816	0.881	0.881	0.899	0.896	0.506	0.501	0.614	0.614	0.662	0.655
3	0.449	0.451	0.549	0.551	0.590	0.581	0.786	0.785	0.830	0.831	0.858	0.856	0.462	0.466	0.549	0.552	0.595	0.587
4	0.356	0.348	0.464	0.461	0.458	0.455	0.708	0.710	0.775	0.780	0.770	0.767	0.374	0.367	0.470	0.465	0.458	0.456
5	0.323	0.322	0.409	0.411	0.395	0.389	0.683	0.681	0.716	0.719	0.719	0.710	0.346	0.345	0.413	0.413	0.397	0.391
6	0.275	0.269	0.338	0.341	0.313	0.308	0.624	0.619	0.645	0.647	0.639	0.634	0.284	0.278	0.343	0.347	0.308	0.300

**Table 10.** Vertical occlusions KL divergences.

Num.	SqueezeNet1_1	ResNet18	VGG16
1	0.015	0.009	0.026
2	0.028	0.014	0.060
3	0.031	0.014	0.064
4	0.026	0.013	0.058
5	0.023	0.011	0.048
6	0.022	0.012	0.041

**Figure 10.**
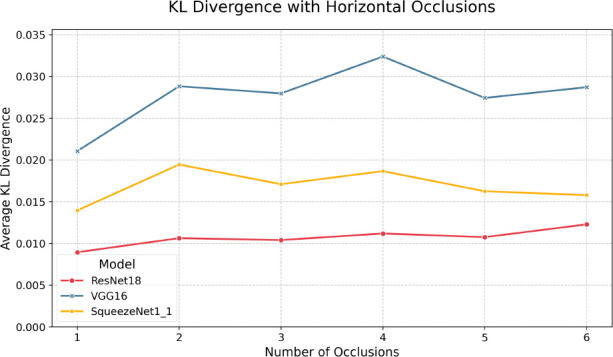
KL divergences for each model pair under various amounts of horizontal occlusion.

Lastly, we look at performance under horizontal occlusions with results shown in Figure 8 and Table 11 while Figure 10 shows the KL divergence. Similar to the results under vertical occlusion, we see an immediate drop-off at just one occlusion, again showing that, generally, models are much more sensitive to highly structured occlusions than AWGN and Brownian noise. Also of note is that VGG-16 performs the best out of all three models across accuracy and F1, unlike the vertical occlusions. F1 is similar to the vertical occlusion results, with a gradual decay congruent with the top-1 accuracy curve. Another interesting note is that we generally observe less degradation for the same number of streaks than vertical occlusions. This may be because the distinguishing features learned during training are dominated by horizontal edges such as the plane’s fuselage, and introducing points of high contrast in the form of structured noise is much more disruptive, as they are guaranteed to intersect the plane’s structure. Further, we see a widened discrepancy between top-1 and top-5 accuracies with horizontal occlusions compared to vertical, suggesting it is likely that generally, models are more likely to have the correct class in their top 5 predictions but struggle with exact classification. This result is likely exacerbated by the dataset’s nature, in that such occlusion severely impairs the model’s ability to distinguish between subtleties in the different classes. We can also see the same behavior as all other studied perturbations with respect to quantization in that there is minimal divergence between the INT8 and FP32 models across all metrics. Most unique to horizontal occlusions is that the KL divergence for all model pairs is near zero and shows no clear trend. This indicates that the output distributions are similar under such perturbation and lack any notable discrepancy.

**Table 11.** Horizontal occlusions KL divergences.

Num.	SqueezeNet1_1	ResNet18	VGG16
1	0.014	0.009	0.021
2	0.019	0.011	0.029
3	0.017	0.010	0.028
4	0.019	0.011	0.032
5	0.016	0.011	0.027
6	0.016	0.012	0.029

## 5. Conclusion

We have compared the robustness of FP32 and post-training INT8 quantized CNNs under input perturbations, ranging from independent and identically distributed AWGN to spatially correlated Brownian noise to vertical and horizontal occlusions. We considered three state-of-the-art models ranging in parameter count, including SqueezeNet1_1, ResNet-18, and VGG-16, and measured raw performance according to F1 score, top-1 and top-5 accuracy. To measure changes in class output confidence pre- and post-quantization, we employed KL divergence. We have found that while degradation was observed amongst all models, substantial degradation in the quantized model *relative* to the full-precision model was not observed. Indeed, we see the highest degree of top-1 and top-5 error between the models in VGG-16 under Brownian noise at σ=20 , and in ResNet-18 under AWGN at σ=0.6 , respectively. We observe the most significant discrepancy in class output probabilities under the Brownian noise regime, followed by AWGN. It is also worth noting that the same presented methodology in terms of examining KL divergence with perturbations can be applied to evaluate the robustness of other model compression techniques, such as approximate multipliers. ^36, 15, 31^

As stated in the introduction, this work sought to address deficiencies in the current body of research surrounding neural network quantization. The goal is to de-risk the deployment of neural networks in real-world scenarios and provide experimental data to understand their performance under various perturbation regimes. Based on these experimental findings, we can conclude that INT8 quantized networks do not exhibit ill-conditioning or exacerbated sensitivity under perturbation relative to their FP32 counterpart and, thus, are robust in these noise regimes.

We have also identified a balance between traditional metrics like top-1 and top-5 accuracy and model similarity. For example, in the AWGN regime, we see that VGG-16 outperforms ResNet-18 but also has a higher KL divergence consistent with lower confidence. Models like VGG-16 also carry a significantly higher energy consumption and increased complexity as measured by MACs and GFLOPs ( Table 1). These results provide a means to guide designers to identify candidate models based on expected deployment scenarios; for instance, if thermal noise is expected, one may consider AWGN, and if similarity in probabilities is more important than top-1 accuracy, ResNet-18, with 8.5x fewer GFLOPs than VGG-16, may be preferred. That is, we have highlighted a crucial trade-off between raw accuracy and KL divergence. We sought not to prove that, for example, VGG-16 is the best performer under perturbation but rather that there are demonstrable trade-offs when considering quantization in terms of accuracy and model similarity under perturbative threats.

## Code availability

Source code available from: https://github.com/jacklangille/CNN-Quantization-Experiment-Code/tree/main

Archived source code at time of publication: https://doi.org/10.5281/zenodo.15097737

License: MIT license
