# Supplementary material for: Quantized Convolutional Neural Networks Robustness under Perturbation
Source: F1000Res. 2025 Apr 9;14:419. [Version 1] doi: 10.12688/f1000research.163144.1 (PMC12041843; doi:10.12688/f1000research.163144.1)
Supplement: Peer review history

# Reviewer report: Reviewer response for version 1

**DOI:** 10.5256/f1000research.179444.r377971

**Published:** 2025-04-29

**Authors:** Venkata Mohit Tamanampudi

**Affiliations:** 1 JP Morgan Chase, New York City, USA

This is a well-executed and clearly written study that fills an important gap in understanding the real-world reliability of quantized CNNs under perturbations. The experiments are thorough, and the conclusions are sound.

 If the authors want to further improve the paper, they could consider:

- Expanding future work discussions around applying the methodology to lightweight and transformer models.
- Discussing how the observed robustness might translate into real-world embedded deployments (e.g., edge devices).
- Publicly releasing trained models and code for even stronger reproducibility, although the current level of detail is already sufficient.

Is the work clearly and accurately presented and does it cite the current literature?

Yes

If applicable, is the statistical analysis and its interpretation appropriate?

Yes

Are all the source data underlying the results available to ensure full reproducibility?

Yes

Is the study design appropriate and is the work technically sound?

Yes

Are the conclusions drawn adequately supported by the results?

Yes

Are sufficient details of methods and analysis provided to allow replication by others?

Yes

Reviewer Expertise:

Artificial Intelligence, deep learning, LLM's, Machine learning, NLP, Devops, AI Agents

I confirm that I have read this submission and believe that I have an appropriate level of expertise to confirm that it is of an acceptable scientific standard.

# Reviewer report: Reviewer response for version 1

**DOI:** 10.5256/f1000research.179444.r380048

**Published:** 2025-04-24

**Authors:** Joseph Chukwunweike

**Affiliations:** 1 Gist Limited, Bristol, UK

The article is technically sound however there are some corrections that needs to be done

 1. References included in the conclusion session should not be included (Removed)

 2. The referencing of the Programme used to run the simulation should be stated.

 3. The references had et al which is not properly structured. if the authors are less than 5, et al cant be used, all the names of the authors should be written.

Is the work clearly and accurately presented and does it cite the current literature?

Yes

If applicable, is the statistical analysis and its interpretation appropriate?

Yes

Are all the source data underlying the results available to ensure full reproducibility?

Yes

Is the study design appropriate and is the work technically sound?

Yes

Are the conclusions drawn adequately supported by the results?

Yes

Are sufficient details of methods and analysis provided to allow replication by others?

Yes

Reviewer Expertise:

Automation, process control, sortation, control, embedded systems, Electronics engineering

I confirm that I have read this submission and believe that I have an appropriate level of expertise to confirm that it is of an acceptable scientific standard.
